# Dry EEG in Sports Sciences: A Fast and Reliable Tool to Assess Individual Alpha Peak Frequency Changes Induced by Physical Effort

**DOI:** 10.3389/fnins.2019.00982

**Published:** 2019-09-20

**Authors:** Selenia di Fronso, Patrique Fiedler, Gabriella Tamburro, Jens Haueisen, Maurizio Bertollo, Silvia Comani

**Affiliations:** ^1^Behavioral Imaging and Neural Dynamics Center, University “G. d’Annunzio” of Chieti–Pescara, Chieti, Italy; ^2^Department of Medicine and Aging Sciences, University “G. d’Annunzio” of Chieti–Pescara, Chieti, Italy; ^3^Institute of Biomedical Engineering and Informatics, Technische Universität Ilmenau, Ilmenau, Germany; ^4^eemagine Medical Imaging Solutions GmbH, Berlin, Germany; ^5^Department of Neurosciences, Imaging and Clinical Sciences, University “G. d’Annunzio” of Chieti–Pescara, Chieti, Italy; ^6^Department of Neurology, Biomagnetic Center, Jena University Hospital, Jena, Germany

**Keywords:** electroencephalography, individual alpha peak frequency, bioelectric activity, sport science, dry electrodes, electrodes, cycling

## Abstract

Novel state-of-the-art amplifier and cap systems enable Electroencephalography (EEG) recording outside of stationary lab systems during physical exercise and body motion. However, extensive preparation time, cleaning, and limited long-term stability of conventional gel-based electrode systems pose significant limitations in out-of-the-lab conditions. Dry electrode systems may contribute to rapid and repetitive mobile EEG acquisition with significantly reduced preparation time, reduced cleaning requirements, and possible self-application by the volunteer but are known for higher channel failure probability and increased sensitivity to movement artifacts. We performed a counterbalanced repeated measure endurance cycling study to objectively validate the performance and applicability of a novel commercially available 64-channel dry electrode cap for sport science. A total of 17 healthy volunteers participated in the study, performing an endurance cycling paradigm comprising five phases: (I) baseline EEG, (II) pre-cycling EEG, (III) endurance cycling, (IV) active recovery, and (V) passive recovery. We compared the performance of the 64-channel dry electrode cap with a commercial gel-based cap system in terms of usability metrics, reliability, and signal characteristics. Furthermore, we validated the performance of the dry cap during a realistic sport science investigation, verifying the hypothesis of a systematic, reproducible shift of the individual alpha peak frequency (iAPF) induced by physical effort. The average preparation time of the dry cap was one-third of the gel-based electrode caps. The average channel reliability of the dry cap varied between 80 ± 15% (Phase I), 66 ± 19% (Phase III), and 91 ± 10% (Phase V). In comparison, the channel reliability of the gel-based cap varied between 95 ± 3, 85 ± 9, and 82 ± 9%, respectively. No considerable differences were evident for the comfort evaluations nor the signal characteristics of both caps. A within-volunteers repeated measure analysis of variance (RM-ANOVA) did not show significant effects of the electrode type on the iAPF [*F*(1,12) = 1.670, *p* = 0.221, $\eta_{\text{p}}^{2}$ = 0.122, Power = 0.222]. However, a significant increase of the iAPF exists from Phase II to Phases IV and V due to exhaustive physical task. In conclusion, we demonstrated that dry electrode cap is equivalent to the gel-based electrode cap based on signal characteristics, comfort, and signal information content, thereby confirming the usefulness of dry electrodes in sports science and other mobile applications involving ample movement.

## Introduction

Electroencephalographic (EEG) measurements are a powerful means for understanding the interrelation of psychology and physiology during physical exercise (di Fronso et al., 2017; Perrey and Besson, 2018). In particular, mobile EEG systems have permitted the collection of brain data in ecological settings, making EEG the most widely used technique to assess brain activity in the context of sports science (Park et al., 2015; Bertollo et al., 2019). EEG monitoring considerably contributed to assess brain functional changes associated with skilled performance in shooting sports (Hatfield and Kerick, 2007; Bertollo et al., 2016), expertise in a juggling paradigm (Filho et al., 2016), and stimuli aiming to ameliorate the effects of fatigue during the execution of isometric tasks (Bigliassi et al., 2016). Other EEG-based studies investigated the modulation of brain activation patterns in different frequency bands due to different attentional strategies and exertion phases, known to influence performance (Comani et al., 2014; di Fronso et al., 2018).

However, the use of conventional stationary EEG setups in sport science is affected by the limited movements allowed when studies on brain activations during physical exercise are performed in ecological conditions. Novel state-of-the-art amplifier and cap systems allow measurements outside the strict laboratory conditions and during ample body movement (e.g., di Fronso et al., 2017), enabling the study of human cognition during natural, realistic ecological conditions. This is an important achievement, as cognitive measures can differ significantly based on body motion and environment, consequently requiring the use of wearable mobile brain/body imaging systems (Makeig et al., 2009; Gramann et al., 2011; Jungnickel and Gramann, 2016). However, the time required for the placement, preparation, and cleaning of conventional, gel-based electrode caps as well as gel drying effects during long-term recordings, still pose significant limitations to the employment of gel-based electrode EEG systems in mobile out-of-the-lab conditions.

Dry electrode caps may contribute to rapid, reliable, and repetitive mobile EEG studies in sport science applications due to decreased preparation effort and time, reduced cleaning needs, and the possibility to be self-applied by the volunteer. However, dry electrodes are known to be more susceptible to movement artifacts and channel failure. Notwithstanding the aforementioned advantages of dry electrodes, it is therefore important to investigate whether dry and gel-based electrodes provide comparable signal quality and outcome of EEG measurements during mobile conditions involving the movement of the user, in order to extract indices of brain activation that can be useful for sport science applications.

During the last decades, the alpha band was recognized to contain the dominant oscillations in the human brain (Klimesch, 2012). Among the measures developed to quantify the amplitude and spectral content of EEG signals within the alpha band and to monitor how they change across experimental conditions (for a review see bazanova and Vernon, 2014), the individual alpha peak frequency (iAPF) has gained increasing attention (e.g., Cheron et al., 2016) because it was demonstrated to account for latent factors of general cognitive abilities (Grandy et al., 2013a). Therefore, iAPF has been increasingly employed as a neural marker of mental stressful conditions in sport science (Matsunami et al., 2001; Lewis et al., 2007; Seo et al., 2008, 2009), and recent studies reported an iAPF increase after acute physical effort induced by an endurance cycling task, speculating that the observed modulation of the iAPF could be related to exercise-induced activation of the brain’s arousal mechanisms and to enhanced alertness during performance (Gutmann et al., 2015, 2018b). Although there have been cases where no significant changes of iAPF were observed when comparing baseline iAPF with pre- and post-task iAPF (Christie et al., 2017), corroborating the notion that iAPF values are invariant (Grandy et al., 2013b), nonetheless the iAPF was suggested as a useful measure to study fatigue and stress-recovery balance (Bertollo et al., 2017) to prevent dysfunctional states such as overtraining and/or injuries in athletes, and to modulate the individual training load in different sports.

Based on the aforementioned observations, it is important not only to demonstrate that dry electrodes are equivalent to gel-based electrodes, with rapid and easy preparation, channel reliability, signal quality, and wearing comfort, but also to verify the occurrence of an iAPF shift (with both electrodes types) that would confirm the usefulness of this measure to asses physical fatigue – a critical parameter for the prevention of overtraining and/or injuries in athletes and for the modulation of the individual training load in different sports. Therefore, we performed a counterbalanced repeated measure study on endurance cycling to: (1) test the performance of a novel 64-channel dry electrode cap in comparison with a conventional gel-based EEG cap system under the same realistic sports science conditions, and (2) verify or reject the hypothesis of a systematic, reproducible iAPF shift consequent to an exhaustive physical task.

Drawing on previous studies (i.e., Fiedler et al., 2015; Gutmann et al., 2015, 2018b), we anticipated that: (1) although channel reliability could decrease for dry electrodes due to some electrode displacements caused by large body movements, the use of a 64-channel cap would ensure that a sufficient number of reliable electrodes will remain to identify iAPF changes related to physical exercise, and (2) an iAPF shift to higher frequencies, detectable with both dry and gel-based EEG caps, would occur in response to intense physical exercise.

## Materials and Methods

### Participants

A total of 17 healthy male volunteers, aged 25.5 ± 4.3 years, participated in the study. All volunteers were selected based on four inclusion criteria: (1) they regularly practiced cycling at least twice a week; (2) they had not reported neurological, psychological, or dermatological diseases; (3) they were not under pharmacological treatment; and (4) their measured head circumference was within the range for the two cap sizes as indicated by the cap manufacturer. The study complied with the ethical standards outlined in the Declaration of Helsinki and was approved by the local institutional Ethics Committee. Prior to study participation, all volunteers provided written informed consent and medical certifications of fitness for participation in non-competitive sports activities.

### EEG Caps

In the present study, we employed dry and gel-based electrode caps with the following common features:

(1) coaxial cables connected directly to the electrodes: This arrangement allowed for application of active shielding, which has been shown to efficiently reduce environmental noise and cable movement artifacts (van Rijn et al., 1990);
(2) two cap sizes (small and medium), which were selected based on head circumference and hair style to optimally adhere to the individual head. The selected cap size per head circumference complied with the recommendations of the cap manufacturer (ANT Neuro b.v., Hengelo, Netherlands).

#### Dry Electrode Cap

The polyurethane-based flexible multipin electrodes with an AgCl coating have been successfully validated in previous studies during lab conditions and the volunteers sitting in a resting, relaxed position (Fiedler et al., 2015).

**FIGURE 1:**
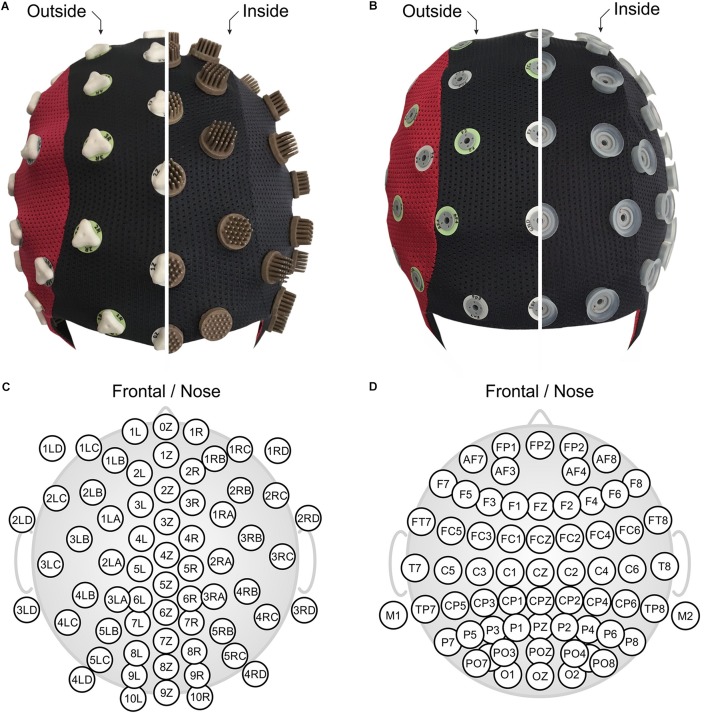
The two compared EEG caps shown turned inside out: **(A)** 64-channel dry PU-AgCl multipin electrode cap with equidistant layout, and **(B)** 64-channel gel-based sintered AgCl electrode cap with extended 10–20 layout. Corresponding detailed 2D electrode topographic layouts are shown in panels **(C,D)** for the dry- and the gel-based caps. Panels **(A,B)** are in front view (nose on bottom), and panels **(C,D)** show nose on top.

In this study, we applied a novel multichannel dry EEG cap (waveguard touch CY-261, ANT Neuro b.v., Hengelo, Netherlands) comprising 64 dry multipin electrodes with shore hardness A98. The used dry electrode cap model differs from previous studies (Fiedler et al., 2015) in terms of (1) channel number (64 vs. 97), (2) improved electrode shape comprising overall 30 pins per electrode (Fiedler et al., 2016), and (3) integration of three different pin lengths in the cap, selective for specific regions of the head, to ensure maximal wearing comfort. All electrodes were integrated into a flexible fabric cap, arranged in an equidistant layout as shown in Figures 1A,C. Two snap fastener leads were integrated into the cap at mastoid positions M1 and M2, respectively, for patient ground and reference electrodes. They allowed application of self-adhesive hydrogel AgCl electrodes (Kendall ECG electrodes H124SG, Covidien LLC, Mansfield, MA, United States), which ensured stable, low-impedance skin-sensor contact. The used equidistant electrode layout is required to ensure a homogeneous flexibility of the cap fabric and consequently homogeneous electrode adduction, which is an important requirement for optimal dry electrode functioning (Fiedler et al., 2016). Furthermore, equidistant layouts are preferred for many state-of-the-art algorithms such as automated artifact correction, source localization, and connectivity analysis (Puce and Hämäläinen, 2017).

Prior to placement of the two self-adhesive patches, the volunteer’s skin was scrubbed with abrasive gel (OneStep Abrasiv Plus, H+H Medizinprodukte, Germany) and subsequently cleaned with ethanol-soaked cotton pads. No impedance threshold was set for the dry electrodes. When mounting the cap, care was dedicated to improving electrode number and stability by visual inspection of the previewed EEG signals.

#### Conventional Gel-Based Cap

The EEG recordings acquired with the dry electrode cap were compared to recordings performed using a conventional gel-based cap (waveguard original CA-208, ANT Neuro b.v., Hengelo, Netherlands). The conventional cap comprised 64 sintered AgCl pin electrodes in a layout based on the extended international 10–20 system for electrode placement (Jurcak et al., 2007) as shown in Figures 1B,D. Reference and patient ground electrodes were integrated into the electrode array at positions CPz and AFz, respectively. After application of the cap to the volunteer head, all electrode cavities were filled with a conductive gel for electrophysiological measurements (OneStep Cleargel, H+H Medizinprodukte, Germany). No intentional skin scratching or abrasion was performed given that this is not required for this type of gel-based cap according to manufacturer indications. The impedance of all electrodes was kept below a threshold of 30 kOhm to comply with current standards in cognitive neurosciences.

### Measurement Setup

Electroencephalography recording sessions with both cap systems (cp., 2.2) were performed sequentially. Head circumference of each volunteer was determined prior to application of the EEG cap to select the correct cap size. The head circumference of all volunteers varied from 54 to 58 cm (56.6 ± 1.3 cm).

All volunteers were asked to wash their hair before the EEG measurements to avoid extensive sweat or grease layers influencing the measurement results. No further skin preparation was performed for the volunteers for either of the two compared caps, except for cleaning the mastoids as described before. This procedure complies with the application recommendations of the commercially available cap systems.

For each volunteer, the two independent recording sessions with one of the two cap types were performed in a randomized sequence that was counter-balanced across all volunteers. An interval of 48 h between the sessions was ensured to allow the volunteer to fully recover from fatigue experienced during the first session (di Fronso et al., 2018).

Each EEG cap was connected to an identical 64-channel referential EEG amplifier for mobile use (eego sports EE-225, ANT Neuro b.v., Hengelo, Netherlands). This amplifier series provides a high input impedance of more than 1 GOhm and supports active shielding of coaxial electrode wires. Previous studies have shown that these conditions enable dry electrode EEG acquisition with increased electrode-skin impedances (Fiedler et al., 2015). All recordings were performed at a sampling rate of 1024 samples/second using the corresponding eego software (ANT Neuro b.v., Hengelo, Netherlands). The recordings were exported in raw, unprocessed condition for further analysis.

A Monark Cycle-Ergometer (939 E, Monark Exercise AB, Vansbro, Sweden), with its power controlled by an external device (Fitmate-PRO, Cosmed, Rome, Italy), was used for four out of the five phases (i.e., from Phase II to Phase V) of the subsequently described paradigm.

### Study Paradigm

The study paradigm was identical for the acquisitions performed with the two cap types and consisted of five sequential phases, characterized by different physical conditions. EEG recordings were acquired during each phase.

The five phases were characterized by the following conditions and tasks:

Phase I (baseline condition): The volunteer was sitting relaxed on a chair without movement for 4 consecutive minutes with 2 min open eyes and 2 min closed eyes.
Phase II (pre-cycling condition): The volunteers were sitting on the cycle-ergometer with their hands on the ergometer’s handlebar. They remained on the cycle-ergometer with closed eyes in a relaxed position without movement. Phase II lasted 2 min.
Phase III (cycling condition): The volunteer performed an endurance task on a cycle-ergometer until exhaustion. A constant cycling rate of approximately 80 revolutions-per-minute (RPM) had to be maintained by the volunteers while the power level of the cycle-ergometer, initially set at 50 W, was increased by 25 W every 2 min. Phase III ended when the volunteer reported maximum perceived sustainable effort, i.e., when their rated perceived exertion (RPE) – considered a reliable index of effort (Impellizzeri et al., 2019) and exhaustion (e.g., Staiano et al., 2018) – was greater than 10 on the CR-10 Borg Scale (Borg and Borg, 2010).
Given our research purposes and based on previous studies (Gutmann et al., 2015, 2018b), we decided to not standardize the duration of Phase III but rather to opt for an endurance cycling task until exhaustion because the latter is instrumental to properly inducing physical effort. Also, this specific task ensures to obtain reliable data during the subsequent paradigm phases (see details below). Reaching exhaustion is an individual process and the duration of Phase III ranged from approximately 6 to 28 min (19 ± 6 min), depending on participants’ physical fitness and expertise (di Fronso et al., 2018).
Phase IV (active recovery condition): The volunteer continued cycling with closed eyes while the power level of the ergometer was set back to 50 W. Phase IV lasted 2 min.
Phase V (passive recovery condition): The volunteer was asked to stop cycling and maintain closed eyes while sitting on the cycle-ergometer. Phase V lasted 2 min.

In addition to the EEG signal quality, channel reliability, and iAPF shift, we evaluated three further parameters to compare the performance of the dry and gel-based caps:

(1) Preparation time: Overall preparation time for each cap was measured from the instant of initial cap placement until the beginning of Phase I.
(2) Wearing comfort: Prior to Phase I and Phase II, and after Phase V, the volunteers were asked to evaluate their individual perceived wearing comfort on a scale ranging from 1 (absolute comfort, no pain) to 10 (maximum imaginable pain) (Scott and Huskisson, 1976).
(3) Impedance: An impedance measurement was performed before and after each phase using the impedance measurement function integrated in the amplifier.

### EEG Signal Processing

Before EEG data processing, all impedance values determined by the EEG amplifier were extracted and analyzed separately without further processing.

Electroencephalography recordings were segmented according to the timing of the recording phases (cp., 2.4). Each segment was visually inspected by two trained EEG experts (double blind check) to identify bad channels, defined as channels exhibiting either a saturated, isoelectric line or a predominantly artifactual, non-physiological signal for >30 s of EEG recording. Bad channels were excluded from further processing.

Further signal processing and analysis was performed using a custom MATLAB algorithm (The MathWorks, Natick, MA, United States). After the application of a bandpass filter (Butterworth, 30th order, cut-off at 1 and 40 Hz), all channels were re-referenced to average reference while excluding the previously identified bad channels. Missing spatial information due to the excluded bad channels was interpolated using spherical spline interpolation in EEGLAB (Perrin et al., 1989). Then, EEG segments were divided into consecutive intervals of 30 s duration, and the power spectral density (PSD) was calculated using the Welch estimation method (Welch, 1967).

The alpha band was defined from 7.5 to 13 Hz (Klimesch, 1999; Gutmann et al., 2015). The iAPF was determined in the mean PSD over all channels by calculating the center of gravity in the alpha band.

Although alpha band power is known to be predominantly increased in the parietal and occipital areas (Klimesch, 1997), Gutmann et al. (2018a) indicated that the use of a region of interest (i.e., subset of electrodes) for the calculation of the iAPF would be too restrictive, suggesting that a whole-head electrode array should be rather employed not only to minimize the influence of inter-individual variability of the areas showing alpha activity, but also to reduce the influence of the electrode placement variability on the results.

We objectively determined the occurrence of considerable alpha activity by setting a threshold to identify the alpha peak value: the threshold was set to be ≥1.3 times the average power of the neighboring theta- and beta-EEG bands. The datasets of four volunteers were not included in the iAPF analysis because no considerable alpha activity was observed in one or more paradigm phases.

### Statistical Analysis

To statistically assess the difference between the iAPF determined using gel-based and dry electrodes during the various paradigm phases, we performed a within-volunteers repeated measure analysis of variance (RM-ANOVA) with a 2 (electrode types: gel-based; dry) × 3 (paradigm phases: pre-cycling; active recovery; passive recovery) design using Bonferroni correction for *post hoc* pairwise comparisons. The sphericity assumption was evaluated using the Mauchly test. Greenhouse–Geisser correction for degrees of freedom was applied in case of non-sphericity. In the ANOVA, effect sizes were calculated using partial eta square (ηp2; Lakens, 2013), for which 0.01, 0.06, and 0.14 are considered small, medium, and large effects, respectively. In the case of multiple comparisons, effect sizes were calculated using the Cohen’s *d* (Cohen, 1988), for which 0.20, 0.50, and 0.80 are considered small, medium, and large effects, respectively. The significance level was set at 0.05 and all statistical analysis was performed using Statistical Package for Social Sciences software (SPSS v. 25, IBM, Armonk, NY, United States).

The statistical assessment was performed for Phase II (pre-cycling condition), Phase IV (active recovery condition), and Phase V (passive recovery condition). We did not include Phase I (baseline condition) and Phase III (cycling condition) in this analysis because they were not of interest for the assessment of iAPF shifts. Moreover, EEG recordings during Phase III (cycling condition) would be noisy due to extensive movement artifacts, therefore likely leading to unreliable results.

## Results

### Preparation Time and Wearing Comfort

The average time for preparation and application of the gel-based EEG cap, including gelling and decreasing all impedances below the threshold of 30 kOhm was 39 ± 18 min. The average time required for the preparation and application of the dry electrode cap was 13 ± 3 min, which is one-third of the conventional cap’s preparation time.

The grand average comfort values reported by the volunteers directly after the cap application was 3 ± 2 for the gel-based cap and 4 ± 2 for the dry cap. After approximately 45 min of overall wearing time (i.e., from the preparation time until the end of Phase II), the reported grand average comfort values did not change. In contrast, after approximately 80 min of overall wearing time (i.e., from the preparation time until the end of Phase V), the reported comfort for both caps had improved and exhibited lower values of 2 ± 2 and 3 ± 2 for the gel-based and dry caps, respectively. The volunteers predominantly reported the frontal and frontal–temporal electrodes to be less comfortable compared to other electrode positions.

After removing the cap at the end of the paradigm, the skin-contact points of both electrode types showed characteristic pressure marks for the silicone rings of the gel-based cap and for the pin tips of the dry electrodes. However, the volunteers did not report any unpleasant skin irritation or long-term effects. All volunteers reported that their skin condition, assessed by visual inspection, recovered during a period of approximately 20 min after the caps (both types) were taken off.

### Electrode Impedance and Channel Reliability

**FIGURE 2:**
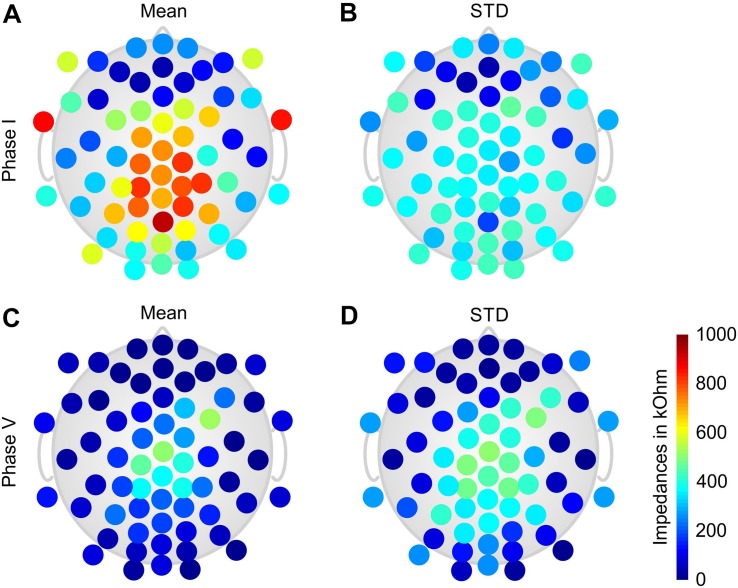
Color-coded topographic plots of the grand average electrode-skin impedances of the dry electrode cap calculated over all volunteers: **(A)** mean and **(B)** STD of the impedances at the beginning of Phase I; **(C)** mean and **(D)** STD of the impedances at the end of Phase V. Orientation of the topographic plot: nose on top.

The grand average electrode-skin impedances of the dry electrodes as determined by the EEG amplifier are shown in Figure 2 at the start of Phase I (Figures 2A,B) and at the end of Phase V (Figures 2C,D). The mean impedance over all electrode positions before Phase I was 455 ± 251 kOhm and had decreased to 132 ± 126 kOhm at the end of Phase V.

The topographic distributions of the grand average impedances show a generally increased impedance level and variability for the central and parietal electrodes. In addition, high impedances are present at the lower frontal–temporal electrodes 1LD/1RD, and 2LD/2RD. Lowest and least variable impedances are observed at the frontal and frontal–temporal electrodes.

The average channel reliability, quantified as the percentage of retained channels after exclusion of bad channels (cp., 2.5), varied considerably during the different phases and conditions. During resting condition Phase I, the mean channel reliability was 95 ± 3% for the gel-based cap and 80 ± 15% for the dry electrode cap. During heavy movement (cycling condition, Phase III) the channel reliability of gel-based and dry electrode caps decreased to 85 ± 9 and 66 ± 19%, respectively. During passive recovery (Phase V), the channel reliability increased back to 82 ± 9 and 91 ± 10%, respectively, for the gel-based and dry electrode caps.

**FIGURE 3:**
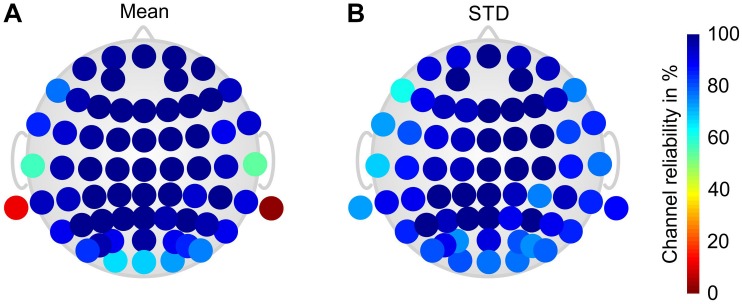
Color-coded topographic plots of the relative channel reliability of the gel-based electrode cap, evaluated by two EEG experts and calculated over all volunteers and phases of the paradigm: **(A)** mean and **(B)** STD. Orientation of the topographic plot: nose on top.

Figure 3 shows the grand average channel reliability of the gel-based electrode cap. The results are calculated over all volunteers and paradigm phases. A considerably reduced channel reliability is visible for the mastoid (M1 and M2) and temporal (T7 and T8) electrodes. Reduced channel reliability is visible also at the occipital and frontal–temporal positions.

**FIGURE 4:**
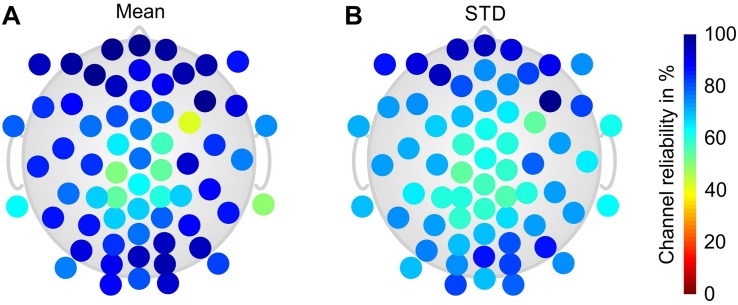
Color-coded topographic plots of the relative channel reliability of the dry electrode cap, evaluated by two EEG experts and calculated over all volunteers and phases of the paradigm: **(A)** mean and **(B)** STD. Orientation of the topographic plot: nose on top.

Figure 4 shows the grand average channel reliability of the dry electrode cap over all phases and volunteers in a color-coded topographic plot. Reduced channel reliability and increased variability are evident at central and parietal head regions, whereas circumferential electrodes exhibit predominantly higher and more stable channel reliability.

### EEG PSD Characteristics

**FIGURE 5:**
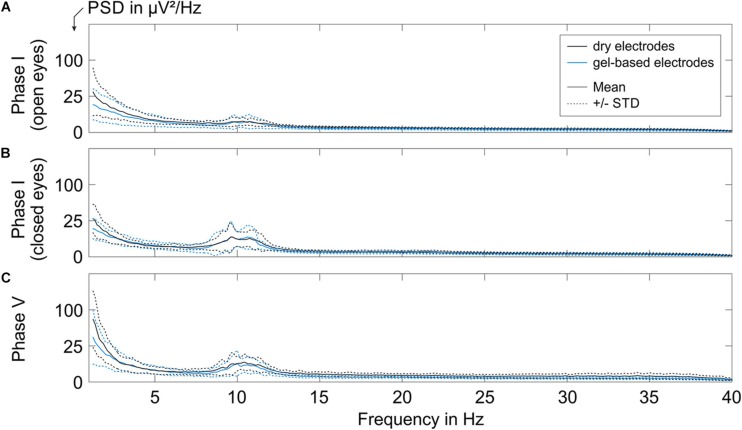
The grand average PSD of the EEG segments recorded with the gel-based and dry electrode caps during **(A)** Phase I with open eyes, **(B)** Phase I with closed eyes, and **(C)** Phase V. Solid lines indicate mean over all volunteers, and dotted lines represent the corresponding STD.

To assess the equivalence of the characteristics of the EEG signals recorded with the dry and gel-based EEG caps, we calculated the grand average PSD over all volunteers during Phase I and Phase V, as shown in Figure 5 (Grand Average ± standard deviation).

A low alpha band power peak was observed for the resting state EEG recorded during Phase I (open eyes sub-condition, Figure 5A) with a mean power of 2.25 ± 1.0 μV^2^/Hz for gel-based and 2.25 ± 0.5 μV^2^/Hz for dry electrode recordings.

The alpha peak power increased during the closed eyes conditions (Phase I, Figure 5B; Phase V, Figure 5C). During Phase I (closed-eye sub-condition), the grand average alpha peak power of the gel-based and dry electrode caps was 7.8 ± 4.8 and 7.8 ± 4.0 μV^2^/Hz, respectively. During Phase V, the grand average alpha peak power of the gel-based and dry electrode caps was 6.25 ± 3.6 and 7.3 ± 1.2 μV^2^/Hz, respectively.

When comparing the mean and STD of the PSD of both electrode types, no considerable difference in spectral signal characteristics could be observed after exclusion of bad channels (cp., 3.2). The order of magnitude of the mean power and STD comply with earlier findings (Fiedler et al., 2015) and are within the range of inter- and intra-individual variability.

### Fatigue-Induced Shift of the iAPF

**FIGURE 6:**
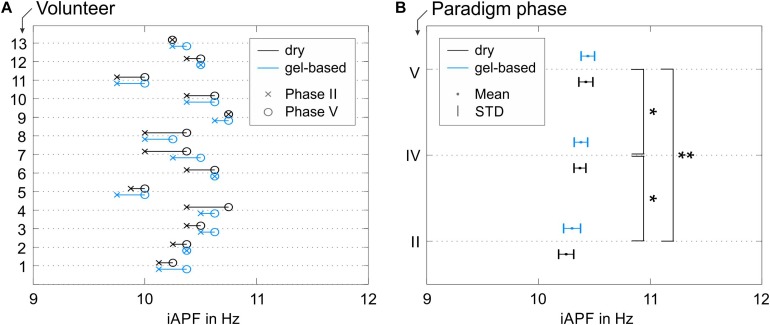
iAPF results for both electrode types: **(A)** individual results in Phase II (pre-cycling condition) and Phase V (passive recovery condition) for all volunteers with alpha activity exceeding the threshold criterion; **(B)** grand average mean and STD calculated over all volunteers in Phases II (pre-cycling condition), IV (active recovery), and V (passive recovery). Asterisks in panel **(B)** indicate significant differences between the iAPF values in the different paradigm phases (^∗^*p* < 0.05; ^∗∗^*p* < 0.001).

The results of the iAPF calculated from the data recorded using both electrode types are shown in Figure 6 (Phases II, IV, and V). The iAPF results are presented only for the 13 volunteers that fulfilled the thresholding criterion of sufficient alpha peak power (cp., 2.5). An increase of the iAPF during the later phases of the paradigm is visible both in the individual iAPF of the volunteers (Figure 6A) and in the grand average calculated over all volunteers (Figure 6B).

Within-volunteers RM-ANOVA did not show significant effects of the electrode type on the iAPF [*F*(1,12) = 1.670, *p* = 0.221, ηp2 = 0.122, Power = 0.222] nor any significant electrode type × paradigm phase interactions [*F*(2,11) = 0.608, *p* = 0.562, ηp2 = 0.009, Power = 0.127]. Consequently, the observed shifts of the iAPF are independent from the electrode type. The observed iAPF effects using the dry electrode cap were systematically equivalent to the effects observed using the conventional gel-based electrode cap.

On the other hand, significant differences between the iAPF calculated for the different paradigm phases were observed [*F*(1.593, 19.113) = 18.665, *p* < 0.001, ηp2 = 0.609, Power = 0.998]. Specifically, *post hoc* pair-wise comparisons with Bonferroni correction showed significant differences between the pre-cycling (Phase II) and the active recovery conditions (Phase IV) (*p* = 0.030, *d* = 0.629), between the pre-cycling (Phase II) and the passive recovery conditions (Phase V) (*p* < 0.001, *d* = 0.819), and between the active and the passive recovery conditions (Phases IV and V) (*p* = 0.037, *d* = 0.232). A constant increase of the average iAPF is evident over the course of Phases II, IV, and V (Figure 6B), which corresponds to a constant fatigue-induced shift of the iAPF toward higher frequencies. Mean and STD of the iAPF values in the different paradigm phases are listed in Table 1 for the two electrodes types.

**TABLE 1:** Mean and standard deviation (STD) of the iAPF values (in Hz) for Phases II, IV, and V of the study paradigm.

**Paradigm phase**	**Condition**	**Gel-based cap**	**Dry electrode cap**
		**iAPF**	**iAPF**
		**(Mean ± STD)**	**(Mean ± STD)**
II	Pre-cycling	10.23 ± 0.30	10.22 ± 0.27
IV	Active recovery	10.37 ± 0.24	10.36 ± 0.21
V	Passive recovery	10.43 ± 0.24	10.41 ± 0.25

*The iAPF values are given for the gel-based and the dry electrode caps.*

## Discussion

We performed a counterbalanced repeated measure study on healthy athletes during an endurance cycling task performed until exhaustion to objectively validate the applicability of a novel 64-channel dry electrode cap for sport applications. We therefore compared the performance of the dry electrode cap with a conventional gel-based cap in terms of preparation time, wearing comfort, electrode-skin impedance, channel reliability, and PSD. Moreover, we investigated whether a systematic, reproducible iAPF shift was observed in the individual volunteers due to physical effort.

During the application and preparation of the gel-based electrode cap, the electrode-skin impedance was decreased below 30 kOhm (cp. 2.2.2). No such threshold could be set for the dry electrode caps because their electrode-skin impedance exhibits a semi-capacitive and strongly varying electrode-skin impedance. In previous studies on dry electrode performance the electrode-skin impedance was reported to depend on contact pressure (Fiedler et al., 2018) and the correlation between high impedance levels and bad channels was found to be low (Fiedler et al., 2015). This implies that, although a low impedance is often associated with a good and stable contact and therefore high signal quality, a high impedance might not necessarily lead to a bad channel. Therefore, our aim during the preparation of the dry electrode cap was to perform a global adjustment of the dry electrode cap to ensure good fit and adduction, followed by a stepwise adjustment and improvement of unstable electrodes based on the observation of signal quality. Adjustment of individual electrodes was therefore limited to those electrodes which initially had a mechanical contact but showed strongly artifactual signals. No further adaptation was performed for electrodes which already showed a stable signal quality or were “floating” (not having skin contact) due to hair underneath. Using this procedure, the average preparation time was measured from the moment of initial cap application to the moment when the electrode skin impedance decreased below 30 kOhm for the gel-based electrode cap, or to the moment when the maximum number of stable channels was achieved with no further possible improvement by manual adjustment for the dry electrode cap. Comparing these conditions between the two cap types, the average preparation time of the dry cap was one-third of the preparation time of the conventional gel-based cap. This result complies with earlier findings and confirms that the use of dry electrodes may facilitate rapid and repeated EEG acquisitions during sports applications.

Wearing comfort was evaluated by asking the volunteers to rate the comfort of the cap after initial application, and approximately after 45 and 80 min of wearing. The exact time of the follow-up comfort evaluations varied across volunteers because of the different duration of the individual Phase III. All volunteers subjectively evaluated the gel-based cap to be slightly more comfortable than the dry electrode cap. However, no substantial difference in the comfort ratings was evident, with the dry electrode cap exhibiting an average comfort rating only one unit higher than the gel-based cap on a scale of 10. In contrast to previous findings (Fiedler et al., 2015) the comfort increased for the majority of volunteers over the course of the paradigm. This effect might be caused by the different mental and physical load and focus of attention of the volunteers. Indeed, the comfort of dry electrode caps was previously validated in a lab-based environment and under resting conditions (Fiedler et al., 2015, 2018). In these cases, the absence of a distinct task might have contributed to direct the focus of the volunteers on the sensations caused by the cap. In contrast, in this study we used a paradigm wherein the volunteers were required to focus on the physical task (i.e., the cycling with increasing power). This specific instruction might have contributed to distracting the volunteers from eventual cap discomfort. When asked about the region of reduced comfort, the volunteers predominantly reported the electrodes in the frontal and frontal–temporal areas to be less comfortable compared to the other electrode positions. This complies well with earlier studies and is likely related to the lower pressure-pain threshold in these areas of the head (Cuadrado et al., 2010). A softer electrode material might help to facilitate a better adaptivity and lower pressure in the frontal–temporal areas of the head, contributing to improve and prolong the overall wearing comfort. The skin marks observed after removal of the caps (both gel-based and dry electrode caps) comply with earlier findings and in both cases the skin of all volunteers recovered during approximately 20 min after taking off the cap. No adverse or long-lasting effects of the skin marks were observed.

Electrode-skin impedance levels and homogeneity have historically been considered as an indicator for the contact reliability and therefore signal quality of gel-based electrodes. However, recent investigations showed that the impact of electrode-skin impedance on signal quality is significantly reduced when using state-of-the-art amplifier technology (Scheer et al., 2006). The initial electrode-skin impedance of the dry electrode cap was considerably higher than in previous studies. This must be ascribed to the different electrode-skin measurement principle of the used amplifier models. While the amplifiers used in our previous studies employed a measurement frequency of 10 Hz, the eego amplifier used here performed a resistance measurement at quasi-DC. The known semi-capacitive electrode–skin interface of dry electrodes exhibits an increasing impedance with lower signal frequencies (Fiedler et al., 2018). The observed increased electrode–skin impedance values are within this range but do not reflect a bad electrode–skin contact quality, as also confirmed by the evaluation of channel reliability. During the course of the paradigm, the impedances of the dry electrode cap decreased to an average of 132 ± 126 kOhm: this reduction is likely due to extensive sweating of the volunteer during physical activity.

Channel reliability was evaluated by two experts based on the visual inspection of signal quality. Given that the EEG signals were acquired using a referential EEG amplifier, channel reliability was influenced by the quality of two electrode contacts: the one of the actually evaluated electrode and the one of the reference electrode. Therefore, cases of generally low channel reliability were essential to be ascribed to a loose contact at the reference electrode. The signal quality reported for the gel-based electrodes was below 100% due to repeatedly failing mastoid and lower temporal electrode positions. Channel quality at these positions was considerably lower, especially during movement, due to a reduced mechanical fixation of these electrodes caused by the fabric of the cap and by the heavy sweating of the volunteers during task performance. The quality of the majority of channels in the gel-based caps was reproducibly very high during all measurements and paradigm phases. The channel quality of the dry electrode cap during resting (Phase I) was 80 ± 15% which complies with previous findings for similar conditions (Fiedler et al., 2015). During heavy movement in the cycling condition (Phase III) the channel reliability of both gel-based and dry electrode caps decreased to 85 ± 9 and 66 ± 19%, respectively: as already known, the dry electrodes were more prone to movement artifacts than the gel-based electrodes. During passive recovery (Phase V), the channel reliability increased back to 82 ± 9 and 91 ± 10%, respectively, for the gel-based and dry electrode caps. In this final phase of the paradigm the channel reliability of the dry electrode caps was for the first time higher than that of the gel-based caps. This fact could be ascribed to two observations: first, gel-based electrodes can undergo gel-running effects due to heavy sweating which may cause gel-bridges across electrodes and thus decrease the signal quality; second, the dry electrodes are not affected by conductive bridges because of the absence of gel. However, the increase of the channel reliability of the dry electrode caps after an endurance task requires further investigation given that extensive sweat may cause bridges between dry electrodes as well.

The spectral signal characteristics of both gel-based and dry electrodes were investigated by quantifying the PSD. Our findings comply with earlier validation results (Fiedler et al., 2015) and do not show considerable differences between the gel-based and the dry electrode caps after exclusion of bad channels: Alpha activity was clearly increased for closed eyes condition (during Phase I and Phase V) compared to open eyes condition (during Phase I). An increased power for frequencies below 3 Hz was evident in Phase V for both electrode types which may be movement-induced or related to sweat or skin-stretching effects. Considering the similar PSD characteristics, we conclude that the two electrode types exhibit equal signal quality and information content.

Besides the technical validation and comparison of the two cap types, we also estimated changes in the iAPF induced by physical effort during an endurance cycling task. We aimed to evaluate iAPF changes obtained with both gel-based and dry electrodes during three different phases of the paradigm (i.e., Phase II/pre-cycling, Phase IV/active recovery, and Phase V/passive recovery conditions). Importantly, no statistically significant differences were found when comparing the iAPF values obtained with gel-based and dry electrodes. Furthermore, no statistically significant interactions could be observed between electrode types and the paradigm phases. This result supports our conclusion that the used electrode type does not affect the outcome of this EEG-based measure.

Moreover, the results pertaining to the iAPF shift toward higher frequencies after the physical effort corroborate previous findings where iAPF significantly increased after an exhaustive exercise (Gutmann et al., 2015). Since iAPF is also considered an index of speed of information processing and arousal, our results may also confirm that an acute bout of exhaustive endurance exercise can activate brain mechanisms supporting information processing and alertness during performance (Klimesch, 1997; Jann et al., 2010; Lambourne and Tomporowski, 2010).

After the exhaustion task, the iAPF values during active recovery condition (Phase IV, the first 2 min of recovery) remained higher than the values observed during the pre-cycling condition (Phase II) and continued to increase in the passive recovery condition (Phase V). These results are in accordance with the findings of Gutmann et al. (2018b), who demonstrated that the iAPF remains elevated for about 30 min after exercise completion. In our study, during active recovery (Phase IV) the volunteers continued to pedal on the cycle-ergometer at a lower power level and completely stopped pedaling only in Phase V. This particular sequence of phases in our study paradigm might have contributed to the further significant iAPF increase during passive recovery.

The physically demanding nature of the endurance cycling task could explain the discrepancy between our findings and those obtained in a recent study on ice-hockey shooting performance (Christie et al., 2017), where the iAPF was found to be invariant and was related to latent cognitive general factors (Smit et al., 2006; Grandy et al., 2013a, b). Indeed, it has been suggested that an iAPF shift occurs only when strong effort, cardiovascular, and metabolic processes are involved (Billiot et al., 1997; Ng and Raveendran, 2007; Gutmann et al., 2015) such as during an endurance cycling task. Consequently, we conclude that the iAPF shifts observed in our investigation are induced by fatigue, hence additionally confirming the usefulness of this measure in the stress-recovery balance assessment (Bertollo et al., 2017).

Considering all the aforementioned results on the iAPF shift, we argue that our hypothesis that a systematic, reproducible iAPF shift occurs as a consequence of physical effort was confirmed. However, further research is needed to better explore the influence of recovery on the iAPF values. Indeed, due to physiological aspects related to the proposed exhaustive task, we could not randomize active and passive recovery phases and consequently we could not assess whether the iAPF shift depended on the type of recovery performed. Future research should hence envisage paradigms and tasks that permit to randomize active and passive recovery or that plan other recovery strategies (e.g., relaxation strategies) in an attempt to establish their impact on the iAPF modulation. It has been suggested that body temperature variations during an exhaustion exercise could also be a physiological cause for the iAPF shift (Nakata et al., 2016; Shibasaki et al., 2016). Given that we did not measure body temperature variations, it remains a speculation, and future studies should consider the relationship among exercise, room temperature, body temperature, and iAPF, as well as the role of other factors potentially contributing to the iAPF shift, such as participants’ expertise and exercise intensity level, different types of populations such as athletes and/or patients. Finally, notwithstanding the statistical robustness of our results – given the observed effect sizes and powers – future studies should engage a larger number of participants to attain more generalizable findings.

## Conclusion

Dry electrodes offer great advantages for the application in sport science compared to conventional EEG techniques. We successfully validated the applicability of a dry electrode EEG cap system for sport science applications by comparing multiple usability metrics and signal quality as well as addressing and verifying the possibility to measure iAPF shifts as a consequence of physical effort.

Our findings demonstrated that no significant differences in signal quality and applicability were observed between dry and gel-based electrodes. Although dry electrodes showed a reduced overall channel reliability and an increased susceptibility to movement artifacts when compared to gel-based electrodes, they have the advantage of allowing a rapid and easy preparation, and can be a choice for sports science or mobile brain–body interaction studies that involve moderate and homogeneous movements (e.g., during normal walking, in neuromarketing applications, or when studying brain–body interaction in virtual environments). The high-density setups employed here enable the compensation of channel-dropouts while providing further spatial information that may be further analyzed in future studies.

Finally, we demonstrated that dry electrodes are equivalent to gel-based electrodes for measures based on PSD and comfort, and that the hypothesis of a systematic, reproducible iAPF shift as a consequence of exhaustive physical exercise was confirmed with both electrode types, thereby confirming the usefulness of dry electrodes in sports science and of measuring iAPF for the assessment of physical fatigue, which is a critical parameter for the prevention of overtraining and/or injuries in athletes, and for the modulation of the individual training load in different sports.

## Data Availability

The datasets generated for this study are available on request to the corresponding author.

## Ethics Statement

The studies involving human participants were reviewed and approved by The Ethics Committee of the University “G. d’Annunzio” of Chieti-Pescara (Italy)(Ethical Application Ref. no. 10-21/05/2015). The patients/participants provided their written informed consent to participate in this study.

## Author Contributions

SF and PF acknowledge equal contributions to the manuscript. SF, GT, and PF performed the data acquisition. GT and PF performed the data processing and analysis. SF and GT performed the statistical analysis. SF and PF wrote the first draft of the manuscript. GT wrote a section of the manuscript. All authors contributed to the conception and design of the study, manuscript revision, and read and approved the submitted version of the manuscript.

## Conflict of Interest Statement

PF is employed by the company eemagine Medical Imaging Solutions GmbH (Berlin, Germany), a sister company of ANT Neuro b.v. (Hengelo, Netherlands). The remaining authors declare that the research was conducted in the absence of any commercial or financial relationships that could be construed as a potential conflict of interest.
